# Prognostic Value of Gamma-Glutamyl Transpeptidase to Lymphocyte Count Ratio in Patients With Single Tumor Size ≤ 5 cm Hepatocellular Carcinoma After Radical Resection

**DOI:** 10.3389/fonc.2019.00347

**Published:** 2019-05-21

**Authors:** Minjun Liao, Wanying Qin, Yan Liao, Renzhi Yao, Junxiong Yu, Weijia Liao

**Affiliations:** ^1^Laboratory of Hepatobiliary and Pancreatic Surgery, Affiliated Hospital of Guilin Medical University, Guilin, China; ^2^Oncology Medical College, Guangxi Medical University, Nanning, China; ^3^Disease Prevention and Control Center of Guilin, Guilin, China; ^4^Department of Anesthesiology, The Second Affiliated Hospital of Guilin Medical University, Guilin, China

**Keywords:** hepatocellular carcinoma, GLR, AFP, survival, prognosis

## Abstract

Prediction of prognosis of hepatocellular carcinoma (HCC) has shown an important role in improving treatment outcomes and preventing disease progression, however, the prognostic indicator of HCC is still lacking. The purpose of this study is to investigate the predictive value of GLR (gamma-glutamyl transpeptidase to lymphocyte count ratio) in single HCC with a tumor size (TS) ≤ 5 cm. A retrospective analysis was performed on 272 patients with TS ≤ 5 cm who underwent radical resection. The Pearson χ^2^ test was applied to discuss the relationship between HCC and GLR, alpha-fetoprotein (AFP). Then univariate and multivariate analysis was utilized to predict the risk factors for survival prognosis in patients. In this study, GLR showed a positive relation with tumor size, tumor-node-metastasis (TNM) stage, microvascular invasion, early recurrence, and serum aspartate aminotransferase (AST) level, while the AFP value only correlated with drinking. Elevated GLR value had poor overall survival (OS) and progression-free survival (PFS) of TS ≤ 5 cm HCC patients, GLR level and tumor size were closely related to the prognosis of small HCC patients compared with AFP. GLR may serve as a prognostic marker for dynamic monitoring of HCC patients with single TS ≤ 5 cm after radical resection.

## Introduction

Hepatocellular carcinoma (HCC) is one of the most common cancers and the major cause of cancer-related death in the world. It accounts for about 50% of the total incidence and mortality of liver cancer in China (1, 2). Guangxi is one of the regions with the highest incidence of HCC in China, accounting for 40% of the total cancer mortality, ranking first in cancer mortality spectrum. Compare to HCC patients with large tumor size (TS), there are more options for treatments of small HCC tumor patients. Resection is still the modality of first choice for the treatment of patients with TS ≤ 5 cm HCC, which has an excellent prognosis after surgery compare to the large HCC (3, 4). Although the diagnosis and treatment of HCC and postoperative management are improving, the long-term prognosis after hepatectomy is still not optimistic due to the high recurrence and metastasis rate of HCC (5). Serum alpha-fetoprotein (AFP) screening has become a routine clinical practice to diagnosis early stage liver cancer and monitoring recurrence in many parts of the developed world (6). However, serum AFP level is a less sensitive and specific indicator in predicting the prognosis of HCC, and has no diagnostic and prognostic value for small HCC (7). Thus, it is urgent to find an effective indicator to predict and surveil HCC.

As we know, the occurrence and development of hepatocellular carcinoma are closely related to inflammatory factors and immune factors (8). In view of the presence of neutrophils, lymphocytes, monocytes, liver enzymes, etc. in peripheral blood, it is easy to obtain the indicators of inflammation, immunity and liver function by using the economic and non-invasive blood biochemical routine. Liver function indicators can directly reflect the state of the liver, and associate with the prognosis of HCC patients. Gamma-glutamyl transferase (GGT) is an enzyme which acts as a marker for variety of cancers such as renal cell carcinoma, breast cancer, lung cancer and ovarian cancer (9–11), and elevated levels of GGT can be a convincing prognostic indicator for the recurrence of HCC after hepatectomy (12, 13). Recently, some researchers have proposed GGT-to-platelet ratio as an independent prognostic predictor for HBV-related HCC overall survival (14, 15). Lymphocytes have been shown to play a key role in anti-tumor in the human immune system, and the infiltration of lymphocytes is usually associated with a better prognosis in cancer, total lymphocyte infiltration has prognostic significance in HCC (16–18). More studies have shown that the neutrophil-lymphocyte ratio could predict prognosis for HCC after hepatectomy (19, 20). Furthermore, some studies have revealed that the ratio of certain indicators is related to the prognosis of HCC, such as AFP to tumor volume ratio, alkaline phosphatase to lymphocyte ratio, aspartic acid to lymphocyte ratio and neutrophil times γ-glutamyl transpeptidase to lymphocyte ratio, etc. (21–24).

In this article, we put forward a model, GLR (gamma-glutamyltransferase to lymphocyte count ratio), which has been shown to be a prognostic marker for nonfunctional pancreatic neuroendocrine tumor patients underwent curative resection (25). The purpose of this study is to demonstrate whether GLR can be used as a prognostic marker for hepatocellular carcinoma. To achieve this goal, we explored the association of GLR with clinical character, overall survival (OS), and progression free survival (PFS) in HCC patients with TS ≤ 5 cm after surgery, to provide guidance for postoperative monitoring and treatment of HCC patients after hepatectomy.

## Materials and Methods

### Study Population

In this study, the specimens and clinical data of 272 HCC patients who underwent hepatic resection were obtained at the Affiliated Hospital of Guilin Medical University, from 1993 to 2011. Patients eligible for the following criteria were enrolled to the retrospective study: (1) a confirmed diagnosis of HCC with single nodule and a diameter < 5 cm; (2) after treatment of radical resection; (3) complete laboratory biochemical data and follow-up records; (4) no lymphatic system disease and blood system disease. Routine examination of these 272 HCC patients, including ultrasonography (US), computed tomography (CT), magnetic resonance imaging (MRI) and blood biochemical examination were performed before the surgery. Data were collected from patients including age, gender, family history, drinking history, smoking history, HBsAg, serum AFP, liver cirrhosis, tumor-node-metastasis (TNM) stage, NEUT, LYMPH, aspartate aminotransferase (AST), alanine aminotransferase (ALT), recurrence, GGT and so on.

### Follow-Up

Long term follow-up was performed in 272 cases of HCC patients after radical resection. In the first 2 years, abdominal ultrasonography, blood routine, liver function index, and serum AFP were performed every 2 months after surgery, and same examinations were conducted every 6 months after 2 years. Overall survival was calculated as the interval between the date of operation and the date of death or the last follow-up date. Progression free survival was calculated as the interval between the date of operation and the date of disease progression or death. Recurrence was defined as the presence of clinical symptoms associated with hepatic ultrasound or serum AFP abnormalities, and the recurrence of HCC within 2 years was defined as early recurrence.

### Statistical Analysis

The data conforming to Gaussian distribution was expressed as mean ± standard deviation (SD) and assessed by independent *t*-test, while categorical data were compared using Chi square (χ^2^) test or the Fisher exact test. Kaplan-Meier survival curve analysis was utilized to compare the survival difference between different HCC groups, and survival distributions were compared with log-rank test. Univariate analysis and multivariate Cox proportional hazards regression model were performed to determine independent prognostic factors. All statistical analyses were performed using SPSS ver. 18.0 (SPSS Inc., Chicago, IL) and *P* < 0.05 were found to be statistically significant.

## Results

### Basic Characteristics of the Study Population

A total of 272 patients (226 males and 46 females) diagnosed with HCC were enrolled in this study. The average age of these patients was 51.90 ± 11.37 years. The mean values of NEUT ( × 10^9^/L), LYMPH ( × 10^9^/L), Platelets (10^9^/L), Albumin (g/L), Globuline (g/L), ALT (U/L), AST (U/L) and ALP (U/L) in these patients were 3.38 ± 1.50, 1.80 ± 0.62, 162.24 ± 72.42, 39.22 ± 4.50, 30.61 ± 5.02, 46.17 ± 44.06, 44.45 ± 41.83, and 87.32 ± 61.22, respectively. And the median of AFP, GGT and GLR were 43.3 ng/ml (range 0.20–9012.00), 44.46 U/L (range 10.84–517.35) and 28.97 (range 4.86–361.75), respectively. Other parameters and more details are shown in Table 1.

**Table 1:** Clinical and biochemical data of examined patients.

**Parameter**	Mean ± SD^*^
Gender: female/male (n)	46/226
Age (years)	51.90 ± 11.37
Family history: present /absent (n)	44/228
Drinking: present /absent (n)	111/161
HBsAg: positive/negative (n)	227/45
Cirrhosis: present /absent (n)	262/10
AFP (ng/ml): median, range	43.3 (0.20–9012.00)
WBC ( × 10^9^/L)	5.86 ± 1.79
NEUT ( × 10^9^/L)	3.38 ± 1.50
LYMPH ( × 10^9^/L)	1.80 ± 0.62
Platelets (10^9^/L)	162.24 ± 72.42
Albumin (g/L)	39.22 ± 4.50
Globuline (g/L)	30.61 ± 5.02
TB (μmol/L): median, range	13.50 (3.66–171.26)
DB (μmol/L): median, range	4.85 (0.5–140.7)
ALT (U/L)	46.17 ± 44.06
AST (U/L)	44.45 ± 41.83
ALP (U/L)	87.32 ± 61.22
GGT (U/L): median, range	44.46 (10.84–517.35)
GLR: median, range	28.97 (4.86–361.75)

* *Data presented as mean ± SD or others*.

*SD, standard deviation; n, number of patients; HBsAg, hepatitis B surface antigen; AFP, alpha-fetoprotein; WBC, white blood cell; NEUT, neutrophil cell count; LYMPH, lymphocyte count; TB, total bilirubin; DB, direct bilirubin; ALT, alanine aminotransferase; AST, Aspartate aminotransferase; ALP, alkaline phosphatase; GGT, gamma-glutamyl transpeptidase; GLR, gamma-glutamyl transpeptidase to lymphocyte count ratio*.

### The Relationship Between Patients' GLR, AFP Level and Clinical Characters

On the base of receiver operating characteristic (ROC) curve analysis, the optimal cut-off value of GLR was 23.3, with a sensitivity of 69.3% and a specificity of 76.6%. The AUC of the GLR was 0.796 [95% confidence interval (CI), 0.756–0.840] for predicting the prognosis in patients with HCC. The relationships between preoperative GLR, AFP level and clinicopathologic parameters of HCC patients with a diameter ≤ 5 cm were analyzed and showed in Table 2. All cases were divided into two groups based on GLR level, 160 patients (58.82%) were identified as high-GLR group with an elevated GLR (>23.3), and 112 patients (41.18%) were identified as low-GLR ( ≤ 23.3) group. High AFP level group (AFP > 200 ng/ml) and low AFP level group (AFP ≤ 200 ng/ml) were also sorted in this study. The results showed that positive relationships were existed between GLR level and tumor size (*P* = 0.038), TNM stage (*P* = 0.006), microvascular invasion (*P* = 0.048), early recurrence (*P* = 0.015), and serum AST level (*P* < 0.001). However, there was no significant relationship between GLR level and gender, age, family history, drinking, HBsAg, cirrhosis, and serum AFP level (all *P* > 0.05). Relatively speaking, the levels of AFP were not significantly correlated with other clinicopathologic parameters (all *P* > 0.05), except drinking (*P* = 0.002).

**Table 2:** Correlation between the clinicopathologic variables and GLR, AFP level in HCC patients.

**Clinical character**	**Variable**	**GLR level**			**AFP (ng/ml)**		
		**≤ 23.3, n (%)**	**> 23.3, n (%)**	***P*-value**	**≤ 200, n (%)**	**> 200, n (%)**	***P*-value**
Gender	Female	24 (52.2)	22 (47.8)	0.096	24 (52.2)	22 (47.8)	0.142
	Male	88 (38.9)	138 (61.1)		144 (63.7)	82 (36.3)	
Age (years)	≤ 55	69 (41.3)	98 (58.7)	0.953	99 (59.3)	68 (40.7)	0.288
	> 55	43 (41.0)	62 (59.0)		69 (65.7)	36 (34.3)	
Family history	Absent	92 (40.4)	136 (59.6)	0.529	143 (62.7)	85 (37.3)	0.461
	Present	20 (45.5)	24 (54.5)		25 (56.8)	19 (43.2)	
Drinking	Absent	74 (46.0)	87 (54.0)	0.053	87 (54.0)	74 (46.0)	0.002
	Present	38 (34.2)	73 (65.8)		81 (73.0)	30 (27.0)	
HBsAg	Negative	21 (46.7)	24 (53.3)	0.413	30 (66.7)	15 (33.3)	0.459
	Positive	91 (40.1)	136 (59.9)		138 (60.8)	89 (39.2)	
Cirrhosis	Absent	7 (70.0)	3 (30.0)	0.119	5 (50.0)	5 (50.0)	0.513
	Present	105 (40.1)	157 (59.9)		163 (62.2)	99 (37.8)	
Tumor size	≤ 3cm	64 (47.4)	71 (52.6)	0.038	83 (61.5)	52 (38.5)	0.924
	> 3cm	48 (35.0)	89 (65.0)		85 (62.0)	52 (38.0)	
TNM stage	I	48 (47.1)	54 (52.9)	0.006	62 (60.8)	40 (39.2)	0.634
	II	50 (46.7)	57 (53.3)		69 (64.5)	38 (35.5)	
	III	10 (24.4)	31 (75.6)		26 (63.4)	15 (36.6)	
	IV	4 (18.2)	18 (81.8)		11 (50.0)	11 (50.0)	
Microvascular invasion	Absent	107 (43.0)	142 (57.0)	0.048	156 (62.7)	93 (37.3)	0.323
	Present	5 (21.7)	18 (78.3)		12 (52.2)	11 (47.8)	
Early recurrence	Absent	88 (45.8)	104 (54.2)	0.015	118 (61.1)	75 (38.9)	0.740
	Present	24 (30.0)	56 (70.0)		50 (63.3)	29 (36.7)	
AFP (ng/ml)	≤ 200	75 (44.6)	93 (55.4)	0.139			
	> 200	37 (35.6)	67 (64.4)				
AST (U/L)	≤ 40	94 (51.1)	90 (48.9)	< 0.001	114 (62.0)	70 (38.0)	0.925
	> 40	18 (20.5)	70 (79.5)		54 (61.4)	34 (38.6)	

*GLR, gamma-glutamyl transpeptidase to lymphocyte count ratio; AFP, alpha-fetoprotein; n, number of patients; HBsAg, hepatitis B surface antigen; TNM, tumor-node-metastasis; AST, Aspartate aminotransferase*.

### Predictors of OS and PFS Using the Survival Cox Regression Analysis

The results of univariate analysis indicated that tumor size (>3 cm), TNM stage (III–IV), microvascular invasion, serum AST (>40 U/L) and (GLR > 23.3) were important factors affecting OS and PFS of HCC. In addition, early recurrence was an important factor affecting OS of HCC (Table 3). After adjusting other positive predictors, the factors mentioned above were assessed using the multivariate Cox regression analysis showed that microvascular invasion (HR = 2.58, 95% CI = 1.70–4.16, *P* < 0.001), early recurrence (HR = 1.76, 95% CI = 1.25–2.55, *P* = 0.001), serum AST > 40 U/L (HR = 1.58, 95% CI = 1.07–2.38, *P* = 0.021) and GLR > 23.3 (HR = 2.13, 95% CI = 1.51–3.05, *P* < 0.001) can play as independent predictors of OS for HCC with a diameter ≤ 5 cm. Moreover, the findings also revealed that those factors include tumor size > 3 cm (HR = 1.55, 95% CI = 1.08–2.23, *P* = 0.019), microvascular invasion (HR = 2.31, 95% CI = 1.60–3.82, *P* < 0.001), and GLR > 23.3 (HR = 2.01, 95% CI = 1.40–2.92, *P* < 0.001) were independent predictors of PFS for HCC with TS ≤ 5 cm (Table 3).

**Table 3:** Analysis Predictors of Overall Survival and Progression-free Survival in Patients with HCC.

**Variable**	**Univariate analysis**			**Multivariate analysis**		
	**HR**	**95% CI**	***P* value**	**HR**	**95% CI**	***P* value**
**OVERALL SURVIVAL**						
Gender (male vs. female)	1.30	0.77–2.21	0.330			
Age, y (> 55 vs. ≤ 55)	1.01	1.00–1.03	0.052			
Family history (present vs. absent)	1.05	0.68–1.63	0.814			
Drinking (present vs. absent)	0.82	0.56–1.20	0.312			
HBsAg (positive vs. negative)	0.75	0.48–1.19	0.226			
Cirrhosis (present vs. absent)	0.76	0.32–1.87	0.556			
Tumor size, cm (>3 vs. ≤ 3)	2.70	1.51–4.81	0.001	1.34	0.93–1.91	0.107
TNM stage (III–IV vs. I–II)	2.40	1.64–3.52	< 0.001	1.37	0.84–2.17	0.081
Microvascular invasion (present vs. absent)	3.38	2.15–5.23	< 0.001	2.58	1.70–4.16	< 0.001
Early recurrence (present vs. absent)	2.65	1.83–3.84	< 0.001	1.76	1.25–2.55	0.001
AFP, ng/ml (>200 vs. ≤ 200)	1.36	0.92–2.00	0.116			
AST, U/L (>40 vs. ≤ 40)	2.06	1.42–2.98	< 0.001	1.58	1.07–2.38	0.021
GLR (>23.3 vs. ≤ 23.3)	2.46	1.69–3.56	< 0.001	2.13	1.51–3.05	< 0.001
**PROGRESSION-FREE SURVIVAL**						
Gender (male vs. female)	1.26	0.74–2.13	0.385			
Age, y (> 55 vs. ≤ 55)	1.43	0.98–2.03	0.057			
Family history (present vs. absent)	1.12	0.72–1.73	0.620			
Drinking (present vs. absent)	0.83	0.57–1.21	0.337			
HBsAg (positive vs. negative)	0.78	0.49–1.23	0.292			
Cirrhosis (present vs. absent)	0.75	0.31–1.85	0.540			
Tumor size, cm (>3 vs. ≤ 3)	2.62	1.57–3.96	< 0.001	1.55	1.08–2.23	0.019
TNM stage (III–IV vs. I–II)	2.37	1.56–3.28	< 0.001	1.23	0.77–1.93	0.327
Microvascular invasion (present vs. absent)	3.12	1.84–4.90	< 0.001	2.31	1.60–3.82	< 0.001
AFP, ng/ml (>200 vs. ≤ 200)	1.28	0.87–1.88	0.203			
AST, U/L (>40 vs. ≤ 40)	2.05	1.38–2.93	< 0.001	1.46	0.98–2.08	0.102
GLR (>23.3 vs. ≤ 23.3)	2.33	1.58–3.41	< 0.001	2.01	1.40–2.92	< 0.001

*HR, hazard ratio; CI, confidence interval; HBsAg, hepatitis B surface antigen; TNM, tumor-node-metastasis; AFP, alpha-fetoprotein; AST, Aspartate aminotransferase; GLR, gamma-glutamyl transpeptidase to lymphocyte count ratio*.

### The Relationship Between the GLR, AFP Level and the Prognosis of Patients

**Figure 1:**
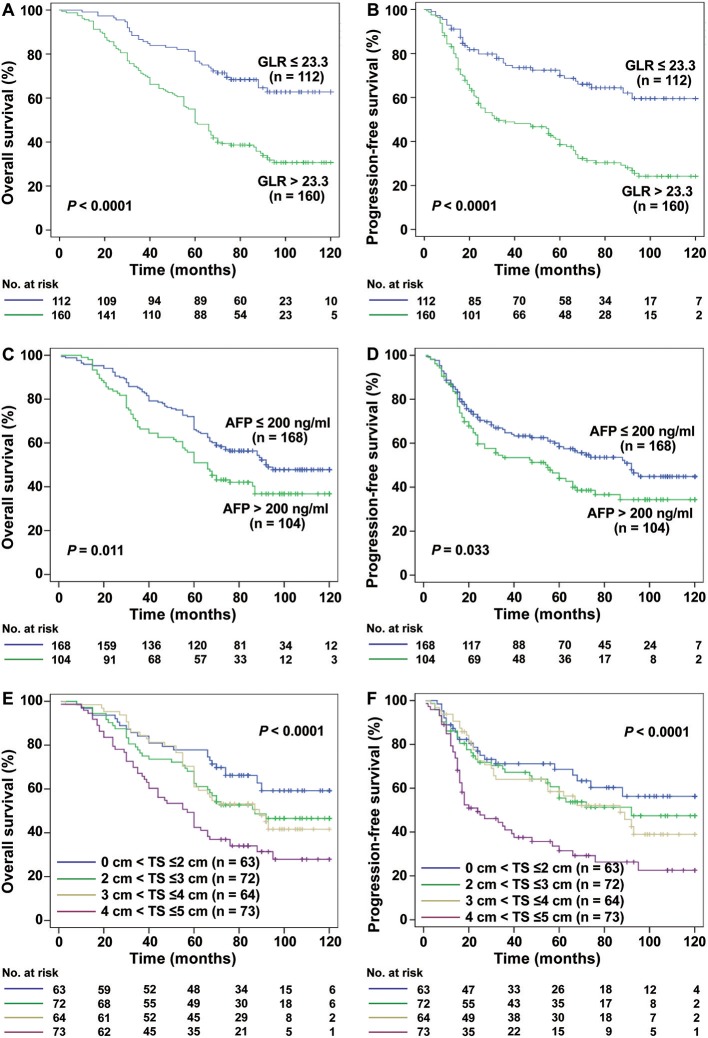
Kaplan-Meier estimated survival curves of HCC (TS ≤ 5 cm) patients after radical resection. **(A,B)** Kaplan-Meier analysis showed that the GLR ≤ 23.3 group (*n* = 112) had a higher rate of OS and PFS than those with GLR > 23.3 (*n* = 160) (both *P* < 0.0001). **(C,D)** The OS and PFS rate of AFP ≤ 200 ng/ml group (*n* = 168) were also higher than AFP > 200 ng/ml group (*n* = 104) (both *P* < 0.05). **(E,F)** In the different tumor size groups, the smaller the tumor size is, the higher the rate of OS and PFS in HCC ( ≤ 5 cm) patients (*P* < 0.0001).

Kaplan-Meier survival analysis showed that the 1-, 3-, and 5-year OS and PFS rates of 272 patients were 93.2, 77.3, and 55.6%; 80.7, 59.3, and 48.5%, respectively. The GLR > 23.3 was significantly associated with poorer OS (Figure 1A) and PFS (Figure 1B). Mean OS was 61.3 months (95% CI, 54.08–68.56) in GLR > 23.3 group and 89.7 months (95% CI, 84.20–95.06) in GLR ≤ 23.3 group (*P* < 0.0001), while mean PFS was 47.12 months (95% CI, 39.21–55.37) and 82.96 months (95% CI, 74.66–90.23) (*P* < 0.0001), respectively. Compared with AFP ≤ 200 ng/ml group, AFP > 200 ng/ml was also associated with poorer OS (*P* = 0.011, Figure 1C) and PFS (*P* = 0.033, Figure 1D).

Furthermore, different TS was also significantly associated with the mean OS (0 cm < TS ≤ 2 cm: 92.38 months, 95% CI, 82.68–102.09; 2 cm < TS ≤ 3 cm: 83.89 months, 95% CI, 74.37–93.42; 3 cm < TS ≤ 4 cm: 81.76 months, 95% CI, 72.48–90.97; 4 cm < TS ≤ 5 cm: 64.95 months, 95% CI, 55.50–74.37; *P* < 0.0001, Figure 1E) and mean PFS (0 cm < TS ≤ 2 cm: 83.02 months, 95% CI, 70.75–95.29; 2 cm < TS ≤ 3 cm: 75.11 months, 95% CI, 63.79–86.40; 3 cm < TS ≤ 4 cm: 71.83 months, 95% CI, 60.52–82.27; 4 cm < TS ≤ 5 cm: 48.31 months, 95% CI, 37.36–59.21; *P* < 0.0001, Figure 1F).

**Figure 2:**
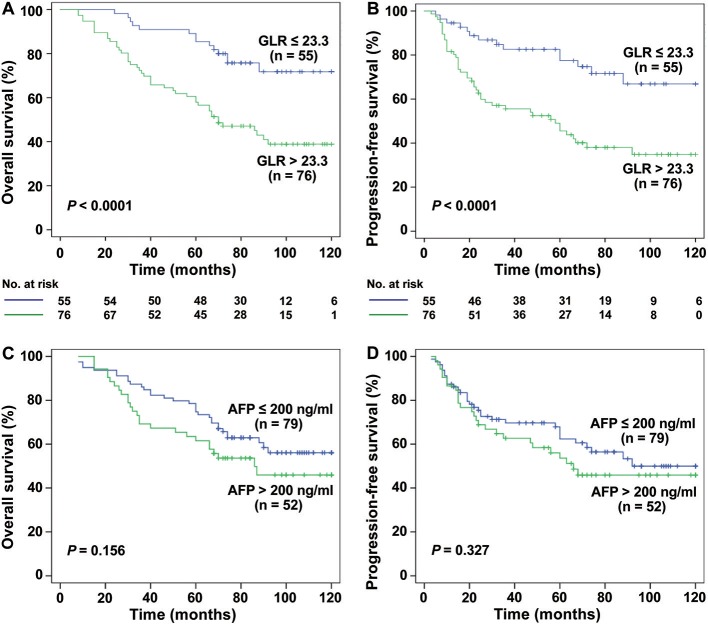
The relation between the GLR, AFP level and the prognosis of TS ≤ 3 cm HCC patients. **(A,B)** Kaplan-Meier analysis showed that the GLR ≤ 23.3 group (*n* = 55) had a higher rate of OS and PFS than the GLR > 23.3 group (*n* = 76) in TS ≤ 3 cm HCC patients (both *P* < 0.0001). **(C,D)** There was no significant difference between the AFP ≤ 200 ng/ml group (*n* = 79) and AFP > 200 ng/ml group (*n* = 52) in the rate of OS and PFS (all *P* > 0.05).

**Figure 3:**
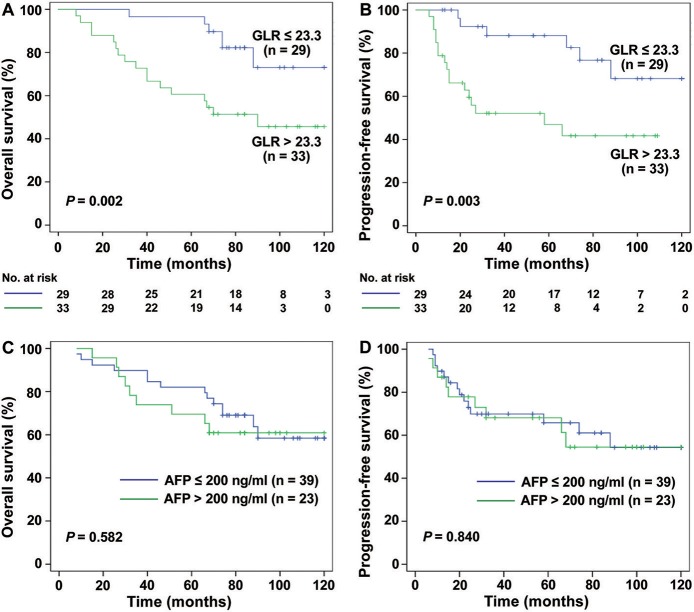
The survival curves of HCC (TS ≤ 2 cm) patients with different levels of GLR and AFP. **(A,B)** The results showed that the rate of OS and PFS in GLR ≤ 23.3 group (*n* = 29) was higher than the GLR > 23.3 group (*n* = 33) in TS ≤ 2 cm HCC patients (*n* = 42) (*P* = 0.002, *P* = 0.001). **(C,D)** The rate of OS and PFS in AFP ≤ 200 ng/ml group (*n* = 39) had no significant difference with AFP > 200 ng/ml group (*n* = 23) (all *P* > 0.05).

Interestingly, for HCC patients with TS ≤ 3 cm (*n* = 131), the mean OS was 64.51 months in GLR > 23.3 group and 95.47 months in GLR ≤ 23.3 group (*P* < 0.0001, Figure 2A), while mean PFS was 51.50 months and 89.51 months (*P* < 0.0001, Figure 2B), respectively. Similarly, the mean OS in GLR > 23.3 group and GLR ≤ 23.3 group were 62.88 months and 99.89 months (*P* = 0.002, Figure 3A), while the mean PFS in GLR > 23.3 group and GLR ≤ 23.3 group were 44.28 months and 91.63 months (*P* = 0.003, Figure 3B) in TS ≤ 2 cm HCC patients (*n* = 62). Conversely, AFP level has no prognostic value in TS ≤ 3 or ≤ 2 cm HCC patients (all *P* > 0.05, Figures 2C,D and 3C,D). These results suggest that GLR level and tumor size were closely related to the prognosis of small HCC patients.

## Discussion

In this study, we compared the relationship between AFP or GLR and clinical characters, OS, and PFS in HCC patients with TS ≤ 5 cm, aimed to validate GLR can serve as an independent prognostic factor of HCC patients after radical resection.

Based on previous studies, the size of tumor has always been one of the most important factors that affect the prognosis of small hepatocellular carcinoma (SHCC) (26). Chen et al. reported that SHCC is defined as a single HCC nodule, which is < 5 cm, and different cut-off values of tumor size may lead to a variety of prognostic significance (27). Nevertheless, the definition of SHCC is based solely on the size of tumor, and it's different from early stage liver cancer which is a concept based on biological behavior (4). And Some SHCC cases may have advanced liver cancer characteristics, which were usually associated with poor prognosis of liver cancer (28), including high TNM stage, vascular invasion, distant metastasis, etc. Of these 272 HCC patients we investigated, there was a certain proportion of TNM III-IV (23%), microvascular invasion (8.5%), and these data confirmed the above point of view (Table 2).

Liver cancer is a typical inflammation-associated cancers, the infiltration of inflammatory cells causes the liver damage by releasing inflammatory factors, and the accumulation of chronic liver injury gives rise to the mutation of liver cells, leading to the occurrence of cancer (29). Globally, 78% of the HCC was caused by HBV (53%) or HCV (25%) chronic infection, which usually accounted for 57% of cirrhosis (30). And our data also supported this conclusion. Of these 272 cases of HCC patients, 227 cases were Hepatitis B surface antigen positive, and 262 cases were liver cirrhosis (Table 1).

Over the years, serum AFP was used as a marker for clinical diagnosis and screening of HCC because of the higher overall survival of patients with preoperative low AFP, and it is believed that AFP is an independent risk factor for postoperative survival (31, 32). However, there was a controversy about the role of AFP in the prognosis of HCC (33). In a survey of 205 patients with SHCC (TS ≤ 3 cm), it was concluded that AFP has no value in predicting the prognosis of SHCC after surgery (7). More studies have also confirmed this phenomenon (34). Similarly, in our results (Figures 1–3), AFP was associated with OS and PFS in these 272 patients with HCC (TS ≤ 5 cm), but there was no prognostic meaning for AFP in the HCC patients with tumor size ≤ 3 cm or ≤ 2 cm. On the other hand, in addition to serum AFP for postoperative monitoring, there are many models or index that can reflect the prognosis of patients with HCC after curative hepatectomy, such as SII (35), GPR (14), NLR (19), etc., and there are some gene expression signatures succeeded in prognosis prediction and treatment responses for HCC (36, 37). However, Gene related prediction models are relatively costly and not conducive to dynamic monitoring dynamic levels *in vivo*, considering the poor specificity and accuracy with single factor to predict the prognosis of SHCC, perhaps we can combine other parameters instead of the single influencing factor to as a prognostic factor for SHCC.

Some studies have reported that vascular invasion was identified as a poor independent prognostic factor for OS and PFS in patients with SHCC, and tumor tissue differentiation was closely related to vascular invasion (38). Our research also proved this point. Other studies have revealed that the disease-free survival rate of the hepatocellular carcinoma ≤ 5 cm without vascular invasion was significantly improved (39). Hence, it is very important for SHCC patients with vascular invasion to closely monitor the situation of early recurrence and take treatment measures to improve the long-term survival rate of patients.

Obviously, there are several limitations in our study. First, the population in our study came from the same third-grade class-A hospital, which will create a selection bias. Secondly, the sample size of the study was too small and had a poor representation and potential bias. Finally, due to the retrospective nature of this study, further prospective with large sample studies are needed to confirm our results.

In conclusion, our findings revealed that GLR was associated with tumor size, TNM stage, microvascular invasion, early recurrence, serum AST level, OS and PFS in the HCC patients with tumor size ≤ 5 cm. GLR can be served as a prognostic marker for < 5 cm HCC patients after radical resection. Therefore, using GLR index to evaluate HCC patients with TS ≤ 5 cm and taking individualized treatment is helpful to improve the long-term prognosis of HCC patients after hepatectomy.

## Ethics Statement

This study was approved by the research ethics committee of the Affiliated Hospital of Guilin Medical University, and the informed consent were obtained from all patients.

## Author Contributions

ML and WQ performed the statistical analysis and wrote the first draft of the manuscript. YL and RY collected cases. JY and WL contributed to the conception and design of the study. All authors listed have made a substantial, direct and intellectual contribution to manuscript revision, read and approved the submitted version.

### Conflict of Interest Statement

The authors declare that the research was conducted in the absence of any commercial or financial relationships that could be construed as a potential conflict of interest.
